# Supplementary figures and images for: Polydopamine-assisted PDGF-BB immobilization on PLGA fibrous substrate enhances wound healing via regulating anti-inflammatory and cytokine secretion
Source: PLoS One. 2020 Sep 29;15(9):e0239366. doi: 10.1371/journal.pone.0239366 (PMC7523965; doi:10.1371/journal.pone.0239366)

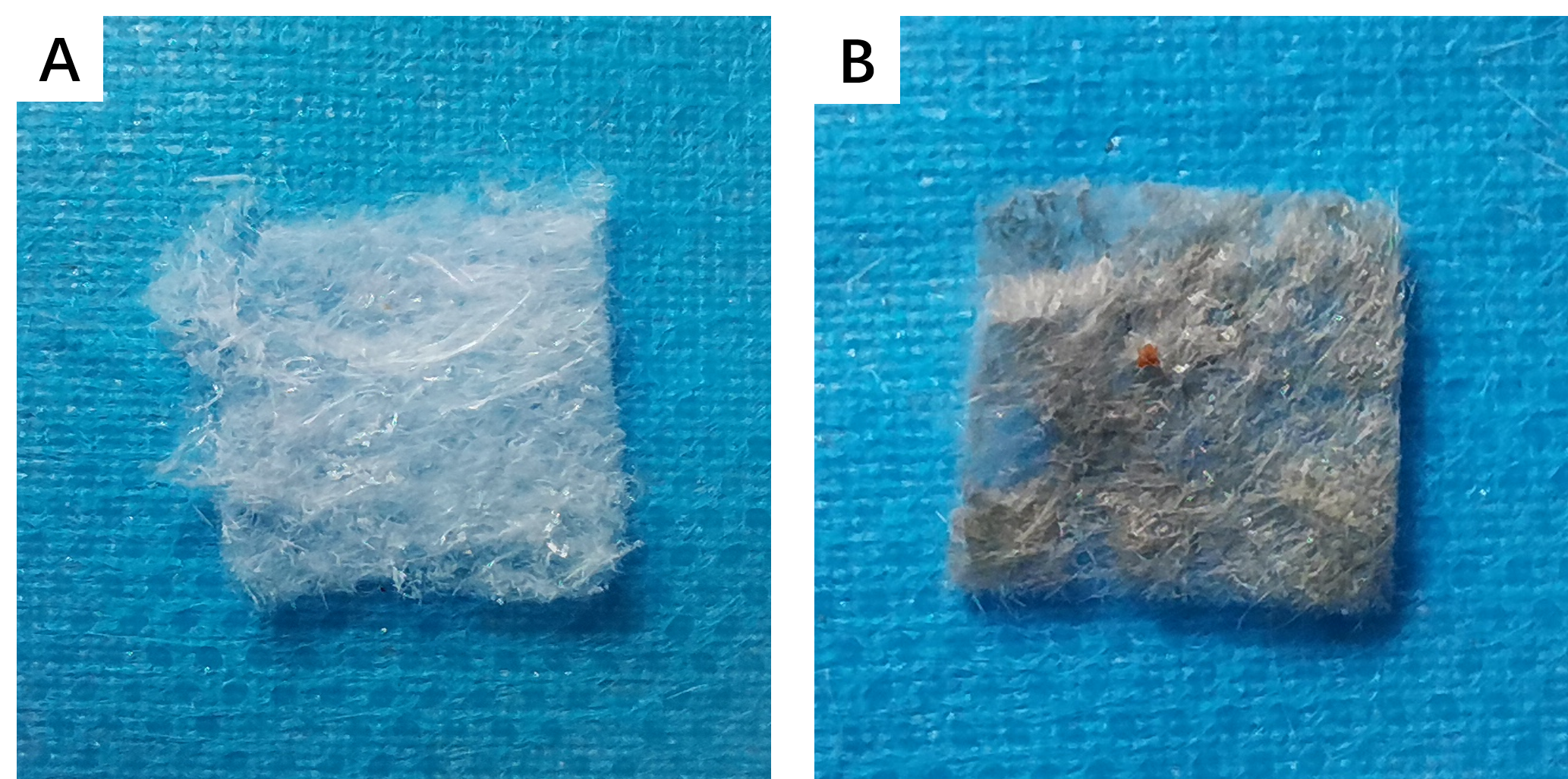

Supplement: S1 Fig — The appearance photographs of (A) PLGA and (B)pDA/PLGA fibers. (TIF) [file pone.0239366.s001.tif]

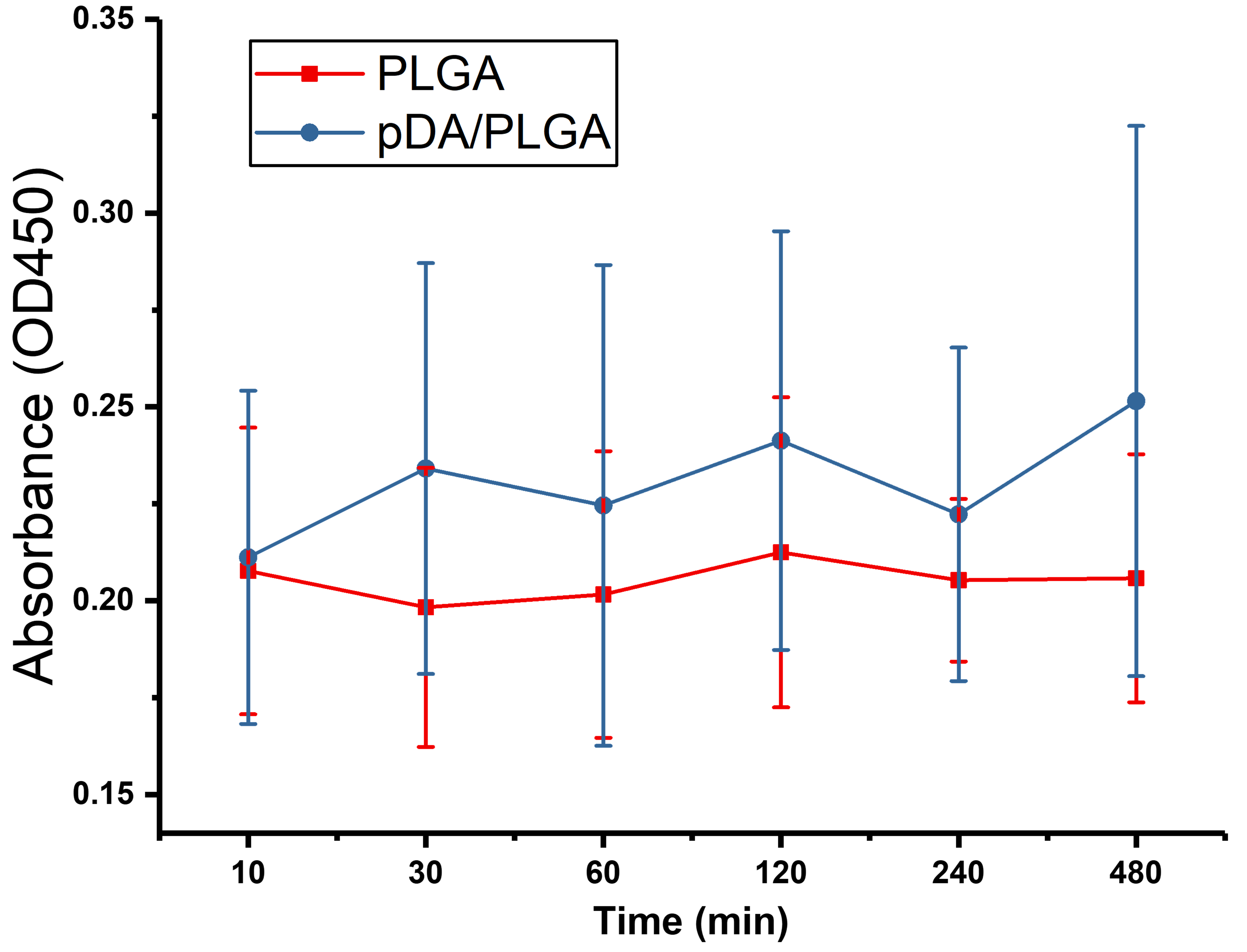

Supplement: S2 Fig — The releasing test of pDA from pDA/PLGA fibers. The PLGA fibers was set as control. All data would be reported in the form of mean±standard deviation. (TIF) [file pone.0239366.s002.tif]

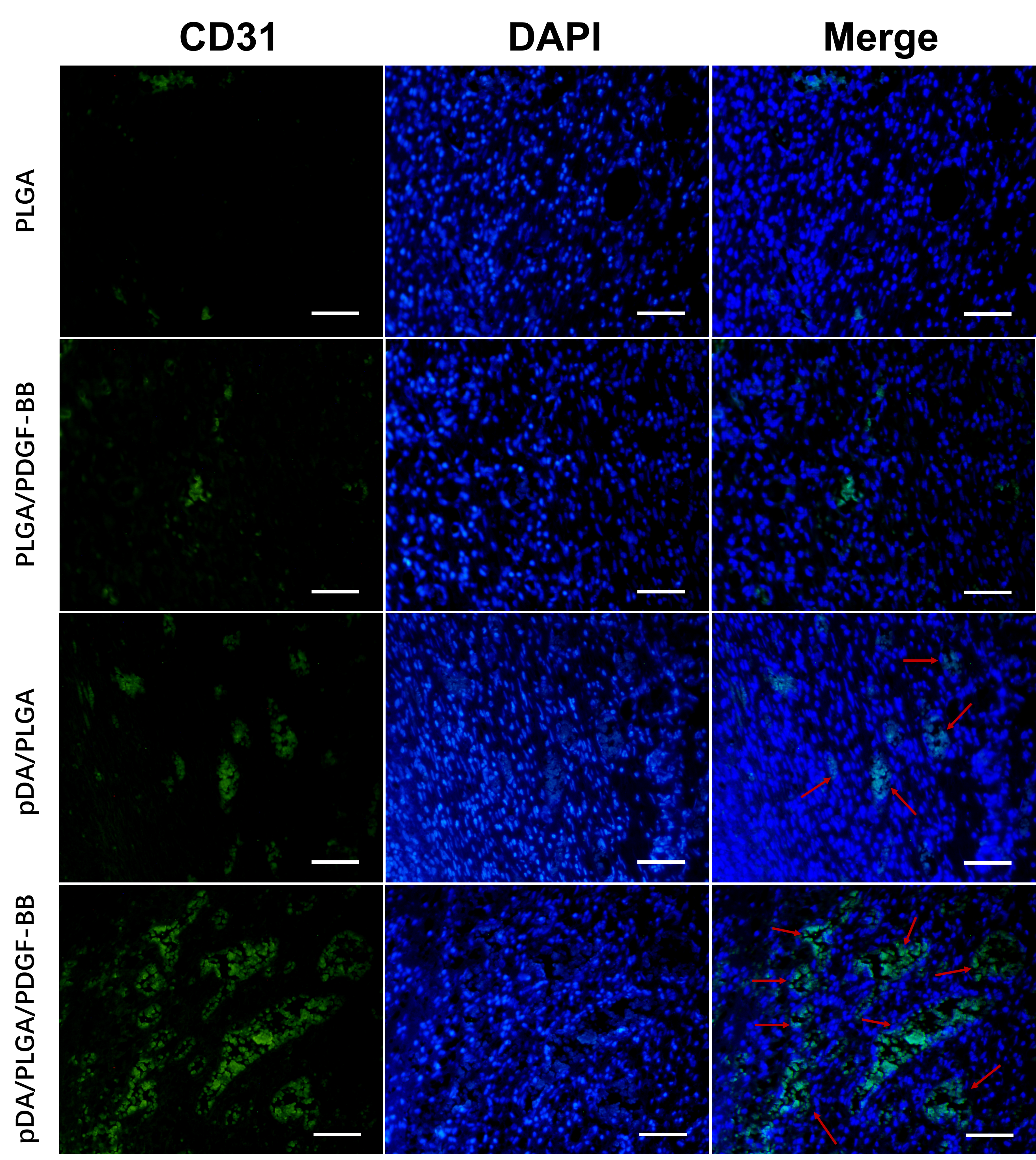

Supplement: S3 Fig — Effects of the substitutes on neovascularization of wound area at day 7 after transplantation by examining the expression of CD31. (The bars = 50 μm). (TIF) [file pone.0239366.s003.tif]
