# Supplementary material for: Polydopamine-assisted PDGF-BB immobilization on PLGA fibrous substrate enhances wound healing via regulating anti-inflammatory and cytokine secretion
Source: PLoS One. 2020 Sep 29;15(9):e0239366. doi: 10.1371/journal.pone.0239366 (PMC7523965; doi:10.1371/journal.pone.0239366)
Supplement: S1 Table — Statistic of fiber diameter of each sample. All data would be reported in the form of mean±standard deviation. (DOCX) [file pone.0239366.s004.docx]

**Table S1. Statistic of fiber diameter of each sample. All data was reported in the form of mean±standard deviation.**

|  | PLGA | PLGA/PDGF-BB | pDA/PLGA | pDA/PLGA/PDGF-BB |
| --- | --- | --- | --- | --- |
| Fiber diameter (μm) | 1.53±0.64 | 1.51±0.71 | 1.61±0.68 | 1.57±0.74 |

Statistic of fiber diameter of each sample. All data was reported in the form of mean±standard deviation.
