# Supplementary material for: Polydopamine-assisted PDGF-BB immobilization on PLGA fibrous substrate enhances wound healing via regulating anti-inflammatory and cytokine secretion
Source: PLoS One. 2020 Sep 29;15(9):e0239366. doi: 10.1371/journal.pone.0239366 (PMC7523965; doi:10.1371/journal.pone.0239366)
Supplement: Attachment — Submitted filename: Responses to Reviewers.doc [file pone.0239366.s006.doc]

**NOTE:** The revised contents in the manuscript were labeled in RED. Our responses were written in BLUE just after each comment (BLACK) of the reviewers. The revised content of manuscript was briefly shown following our response.

First of all, we would like to express our great appreciation to the reviewers for their helpful comments and suggestions.

Journal Requirements:

When submitting your revision, we need you to address these additional requirements.

**1. Please ensure that your manuscript meets PLOS ONE's style requirements, including those for file naming. The PLOS ONE style templates can be found at**

**http://www.journals.plos.org/plosone/s/file?id=wjVg/PLOSOne_formatting_sample_main_body.pdf and http://www.journals.plos.org/plosone/s/file?id=ba62/PLOSOne_formatting_sample_title_authors_affiliations.pdf**

**Response:** Thank you for your kind comments. The manuscript has been revised to meet PLOS ONE's style requirements.

**2. At this time, we request that you please report additional details in your Methods section regarding animal care, as per our editorial guidelines: 1) Please provide details of animal welfare (e.g., shelter, food, water, environmental enrichment, source of animals) 2) please describe any steps taken to minimize animal suffering and distress, such as by administering analgesics, 3) please include the method of sacrifice and 4) Please describe the post-wound care received by the animals, including the frequency of monitoring and the criteria used to assess animal health and well-being. Thank you for your attention to these requests.**

**Response:** Thank you for your kind comments.

Page 9-10：……All animal studies were conducted in accordance with the principles and procedures outlined in “Regulations for the Administration of Affairs Concerning Lab-oratory Animals”, approved by the National Council of China on October 31, 1988, and “The National Regulation of China for Care and Use of Laboratory Animals”, promulgated by the National Science and Technology Commission of China, on November 14, 1988 as Decree No. 2. Protocols were approved by the Committee of Jilin University Institutional Animal Care and Use. Healthy 8-week-old Sprague Dawley (SD) male rats (purchased from Changchun Institute of Biological Production) weighing ~200g were settled in Experimental Animal Center of Jilin University. Adequate supply of food and water was provided. The rats were randomly divided into four groups of four animals each, and anesthetized by i.p. injection of pentobarbital (40 mg/kg). The middle back area was shaved and disinfected with 75% alcohol. Then, two full-thickness orbicular wounds (diameter of 1.5 cm) were created on each side of their dorsum. Then, different substrates were cut into a suitable size and implanted onto the wounds, followed by injection of penicillin sodium for 4 days continuously. The wounds were covered with sterilized double-layer gauze. After surgery, the animals were placed in separate cages and nourished by assigned person. On day 7 postsurgery, the photographs of wounds were taken. The reduction in the size of wounds was determined from macroscopic images and by estimating the wound area using ImageJ software. ……

**Reviewer #1:**

**1. It seems a real control untreated group of animals is lacking; Can you explain why?**

**Response:** Thank you for your kind comments. The regeneration materials based on PLGA nanofibrous substrate has been widely reported. However, the lack of bioactivity was still a burning problem. This study focused on the enhanced bioactivity of PLGA substrate profited from the polydopamine-assisted PDGF-BB immobilization. An untreated control group was surely useful to show the natural repair of wound. Nevertheless, in order to extrude this emphasis, the PLGA group was set up as control. The results also proved that the ability of PLGA substrate on skin repair was significantly improved by polydopamine-assisted PDGF-BB immobilization.
**2.Figure 6: authors claim that the arrows indicate the border between regenerated tissue and normal tissues. Although this is not clearly visible, wound healing at 7 days should be in the proliferative phase and not in the absence of "regenerated" tissue. "Regenerated" is not a term that can be used in skin wound healing: skin repairs, does not regenerate. Can you clarify?**

**Response:** Thank you for your kind comments. In this part, the main task was to point out the positive effect of polydopamine-assisted PDGF-BB immobilization on fusion between newborn and original skin. Actually, according to the results showed in Figure 6, after treatment with pDA/PLGA/PDGF-BB, the border between proliferative tissues and original tissues was ambiguous, while visible border could be observed in other groups. The borders in groups of PLGA/PDGF-BB and pDA/PLGA were not as clear as that in PLGA group. This result indicated that the PDGF-BB and pDA both could improve fusion between proliferative and original skin. Detailed description of these results was added in the manuscript. However, your comments made me realize that the description was incorrect. Thank you for your correction and we have revised it in the manuscript according to the comments.

**Page 13:** “……After 7 days, the damaged dermal tissue area was still large for the untreated. Actually, according to the results showed in Figure 6, after treatment with pDA/PLGA/PDGF-BB, the border between proliferative tissues and original tissues was ambiguous, while visible border could be observed in other groups. The borders in groups of PLGA/PDGF-BB and pDA/PLGA were not as clear as that in PLGA group. This result indicated that the PDGF-BB and pDA both could improve fusion between proliferative and original skin. Particularly, according to……”

**3.Figure 7: blood vessels are not visible. The antibody seems to be unspecific, please provide high magnification where endothelial cells are stained.**

**Response:** Thank you for your kind comments. Anti-CD31 antibody is commonly used in most histopathology laboratories as a vascular marker and is thus readily available to most histopathologists. During routine practice it became apparent that CD31 was also a good marker of microthrombus in tissue sections. And the microthrombus was quite common in post-mortem tissue and located in some mature vessels. In Figure 7, a lot of granulated positive staining was observed in group of pDA/PLGA/PDGF-BB, which was believed to be microthrombus. These microthrombi were located in the lumen of staining-positive cells. Moreover, the presence of the microthrombi indicated that the vessels were mature enough to take on more blood supply. In order to make this part more clear, the images were updated. And the neovessels were point out with arrows in merged images. In addition, the manuscript was also revised.

Page 13: ……Particularly, according to the result of CD31 staining (*Figure 7*), a lot of granulated positive staining was observed in group of pDA/PLGA/PDGF-BB, which was believed to be microthrombus. These microthrombi were located in the lumen of staining-positive cells. PDA/PLGA/PDGF-BB substrate treated group showed a highly angiogenesis structure, which might be attributed to the facilitated penetration of PDGF-BB into deep dermal tissues and the prolonged residence of PDGF-BB conjugate……

Page 14：……But PDGF is particularly important in blood vessel maturation. During routine practice it became apparent that CD31 was also a good marker of microthrombus in tissue sections. And the microthrombus was quite common in post-mortem tissue and located in some mature vessels. The presence of the microthrombi indicated that the vessels were much more mature to take on more blood supply in group of pDA/PLGA/PDGF-BB. In *vivo* experiments have shown that PDGF is critical for the recruitment……

Reviewer #2:

The authors investigate the immobilization of Platelet-derived growth factor-bb on the surface of PLGA electrospun fibers, aiming at controlled growth factor delivery to stimulate wound healing in full thickness wounds in rats. To deliver on this, a polydopamine coating is attempted on the fibers. My main concerns are around the material design and characterization, specifically:

**1) The data presented do not fully support the formation of a polydopamine coating on the fibers; can the authors carry out some degradation experiments to check that no dopamine leaches out from the fibers? At the moment, the data supports that there is deposition of dopamine species, yet the formation of the polydopamine coating is not fully demonstrated. The authors should provide convincing evidence for this claim.**

**Response:**

Thank you for your kind comments. In order to prove the polydopamine coating on the fibers, the appearance photographs of PLGA and pDA/PLGA fibers were taken. As shown in Figure S1, the pDA/PLGA fibers were dark brown in appearance, while the PLGA fibers were yellowish. It was due to the dark color of dopamine. In order to remove free dopamine, the pDA-coated nanofibers had been adequately washed with deionized water five times. Therefore, the dopamine was considered to be stability on the fibers. Even though a little of the dopamine would still fall off the materials.

Figure S1. The appearance photographs of (A)PLGA and (B)pDA/PLGA fibers.

Page 8: ……In order to prove the polydopamine coating on the fibers, the appearance photographs of PLGA and pDA/PLGA fibers were taken. The microstructure images of the membranes were obtained by an environmental scanning electron microscope (ESEM, XL30 ESEM-FEG, FEI).……

Page 10：……As shown in Figure S1, the pDA/PLGA fibers were dark brown in appearance, while the PLGA fibers were yellowish. It was due to the dark color of dopamine. In order to remove free dopamine, the pDA-coated nanofibers had been adequately washed with deionized water five times. Therefore, the dopamine was considered to be stability on the fibers. Even though a little of the dopamine would still fall off the materials.……
**2) What is the mechanism of growth factor immobilization and what is** **the** **kinetics of growth factor release? The authors should provide data on this using controls made doped with the growth factor in the absence of the dopamine treatment. These data would also help in explaining some of the results obtained with the study in vivo.**

**Response:** Thank you for your kind comments. the mechanism of growth factor immobilization has been clarified in the part of introduction. Under alkaline conditions, a poly(dopamine) layer is formed on materials through the catechol groups. And the dopamine may offer a lot of activated amino and phenyl to immobilize peptides and proteins through hydrogen-bond interaction.

In order to clarify the kinetics of growth factor absorption and release on PLGA fibers, the [quantitative](javascript:;) absorption and release tests based on ELISA method were conducted. Related content was added in revised manuscript.

Page 8:……The substrates were washed with deionized water three times to remove the free PDGF-BB. The PLGA without pDA coating was also set up to adsorb the PDGF-BB as contrast.

The quantitative absorption and release tests based on ELISA method were conducted as follow. When PDGF-BB was absorbed on to PLGA and pDA/PLGA fibers as described above, 100 μL supernatant was collected as sample at each time point. After incubation and wash, the fibers were directly soaked into 3 mL PBS (pH 7.2). 100 μL supernatant was collected as sample at each time point. All samples were diluted according to the range of ELISA kit., and detected quantificationally using the PDGF-BB DuoSet ELISA Development kit (R&D system). The absorption rate and release rate were calculated according to following formula:

Absorption rate (%) = (Initial Amount-Sample Amount)/Initial Amount × 100%

Release rate (%) = Sample Amount/ Absorption Amount × 100%

……

Page 13: ……

3.2 Kinetics of growth factor absorption and release on PLGA fibers

The kinetics of growth factor absorption and release on PLGA fibers were detected. As shown in *Figure 5(A)*, At each indicated point, the absorption rate of PDGF-BB on pDA/PLGA was significantly higher than that on PLGA (*p* < 0.01), indicating that the retained PDGF-BB on pDA/PLGA was more than that on PLGA. In the in *vitro* release experiment [*Figure 5(B)*], sustained release of PDGF-BB on pDA/PLGA and PLGA was followed up to 120 mins. PDGF-BB quickly released from materials at the first 20 mins. After that, the PDGF-BB continued releasing slowly from PLGA fibers, whereas it could be sustained on pDA/PLGA from the 20 minutes to 120 minutes. During 120 minutes, the release rate of LBD-BDNF on on pDA/PLGA was significantly higher than that on PLGA (*p* < 0.01). Therefore, pDA/PLGA fibers could effectively absorb PDGF-BB, keep PDGF-BB retaining on and sustained releasing from substrate in *vitro*.……

**3) The SEM images show the internal structure of the fibers. The Authors should assess the average fiber diameter for each sample in order to provide quantitative results. Why do the fibers of the growth factor-loaded scaffolds following dopamine treatment look thicker than all the other sample groups?**
**Response:** Thank you for your kind comments. In the supporting information of revised manuscript, a table containing the average fiber diameter for each sample was given (Table S1.). The result was a statistic based on at least 5 photos from each sample. However, there was no significant difference between samples. Many factors could influence the morphology of fibers. And the voltage during preparation was sometimes fluctuant slightly. So that the morphology of fibers on the same sample might be non-uniform to a certain degree. The SEM images shown in the original manuscript might be misleading. The image of the growth factor-loaded scaffolds following dopamine treatment has been replaced by another image of the same sample.

Fig 5. The quantitative (A)absorption and (B)release tests based on ELISA method. **p<0.01

Page 8: ……The microstructure images of the membranes were obtained by an environmental scanning electron microscope (ESEM, XL30 ESEM-FEG, FEI). Fiber diameter for each sample was measured and recorded using ImageJ. The average fiber diameter was a statistic based on at least 5 photos from each sample. Fourier transform infrared spectroscopy (FT-IR, Perkin Elmer, FTIR-2000) was used to determine the chemical structure of the membranes.……

Page 11:……It may be ascribed to the nHA in Wang’s material. The average fiber diameter for each sample was given in Table S1. The morphology of fibers on the same sample might be non-uniform to a certain degree. It was because that many factors could influence the morphology of fibers. And the voltage during preparation was sometimes fluctuant slightly. However, there was no significant difference between samples. ……

S1 Table. Statistic of fiber diameter of each sample. All data would be reported in the form of mean±standard deviation.

|  | PLGA | PLGA/PDGF-BB | pDA/PLGA | pDA/PLGA/PDGF-BB |
| --- | --- | --- | --- | --- |
| Fiber diameter (μm) | 1.53±0.64 | 1.51±0.71 | 1.61±0.68 | 1.57±0.74 |

**4) The authors attribute the increased wound closure of the wounds in receipt of the growth factor loaded and dopamine treated scaffolds to the prolonged PDGF therapeutic effect, due to the effect of the immobilization. How is this immobilization achieved and can the authors support this with release data in vitro?**
**Response:** Thank you for your kind comments. the mechanism of growth factor immobilization and the release data in vitro has been given under the comment 2. I hope that the response was clear to answer the questions.
**5）The manuscript also lacks a clear motivation as to why this study is original. The authors state in the introduction that growth factor immobilization has already been reported. What is the rational for this study then?**

**Response:** Thank you for your kind comments. As introduced in manuscript, the Polydopamine-assisted growth factor immobilization has been used in many sections of tissue engineering. The materials modified with different growth factors may get various bioactivities. So that exploring the specific bioactivity of each material modified with different growth factors is quite valuable and interesting. In this research, the pDA-assisted immobilization method was originally applied on bonding between PDGF-BB and PLGA fibrous substrate for in *vivo* wound healing. Furthermore, this report instructively confirmed the reduction of TNF-α and increasing of TGF-β and VEGF caused by the long-term effect of the conjugated PDGF-BB on pDA/PLGA/PDGF-BB substrate. These results would be helpful to illuminate the underlying mechanism in subsequent studies.

Page 6: ……The materials modified with different growth factors may get various bioactivities. So that exploring the specific bioactivity of each material modified with different growth factors is quite valuable and interesting. In this research, the pDA-assisted immobilization method was originally applied on bonding between PDGF-BB and PLGA fibrous substrate for in *vivo* wound healing. In this study, we chose the PLGA……
