# Supplementary material for: Polydopamine-assisted PDGF-BB immobilization on PLGA fibrous substrate enhances wound healing via regulating anti-inflammatory and cytokine secretion
Source: PLoS One. 2020 Sep 29;15(9):e0239366. doi: 10.1371/journal.pone.0239366 (PMC7523965; doi:10.1371/journal.pone.0239366)
Supplement: Attachment — Submitted filename: Responses to Reviewers.doc [file pone.0239366.s007.doc]

**NOTE:** The revised contents in the manuscript were labeled in RED. Our responses were written in BLUE just after each comment (BLACK) of the reviewers. The revised content of manuscript was briefly shown following our response.

First of all, we would like to express our great appreciation to the reviewers for their helpful comments and suggestions.

**Reviewer #1: Dear authors, thank you for the provided responses. However it is my opinon that green staining that is seen in figure 7 depends on red blood cell unspecific fuorescence. without high magnification images I cannot say that this is acceptable.**

**Response:** Thank you for your kind comments. The images were updated into high magification. And the positions of vascularization and microthrombus were point out with red arrows in merged images.

**Reviewer #2:**

**1.The authors have clarified the points raised previously with additional results that support their claims. The question on the stability of the coating is still only partially answered and it would need an additional degradation test to check the kinetics of any dopamine released. I would invite the authors to do that. On the in vivo study, the authors may also discuss the results in the context of the literature, looking e.g. at J. Mater. Chem. B 2016 (4) 7249-7258.**

**Response: Thank you for your kind comments. The articles you recommended inspired me a lot.** **The release of drug is often accompanied by material degradation. However, the situation was different in the polydopamine-assisted system. The coating of polydopamine on materials was formed by the covalent bonds and strong noncovalent forces, including hydrogen bonding, charge transfer, and π-stacking. Polydopamine would usually undergo degradation in vitro in the presence of oxidizing agents such as H_2_O_2_. In vivo studies performed by Langer’s group have also demonstrated that It took 8 weeks for polydopamine implants to completely degrade. It is quite a long time to wound healing. In order to demonstrate** **the** **stability of the coating, a degradation test was conducted as described in manuscript. And the result was shown in Figure S2.**

**S2 Fig. Stability of coating polydopamine.** The releasing test of pDA from pDA/PLGA fibers. The PLGA fibers was set as control. All data would be reported in the form of mean±standard deviation.

**Page 7：** ……In order to demonstrated the stability of the polydopamine coating, a test was conducted. The PLGA and pDA/PLGA fibers were respectively soaked in 3 mL PBS solution (pH 7.2). 100 μL supernatant was collected as sample at each time point and detected the absorbance at 450 nm using an auto microplate reader (Tecan M200, Switzerland) as reported.[23]……

**Page 13-14：**……The stability of the coating polydopamine was test. The result was shown in Figure S2. The released dopamine will form nanoparticles in aqueous solution, turning the solution to brown. The color changing can be semiquantitatively detected under OD 450. According to the result, there is no significant difference between PLGA and pDA/PLGA fibers. It indicated that dopamine barely releases from the PLGA fibers. The release of drug is often accompanied by material degradation.[24] However, the situation was different in the polydopamine-assisted system. The coating of polydopamine on materials was formed by the covalent bonds and strong noncovalent forces, including hydrogen bonding, charge transfer, and π-stacking.[25] Polydopamine would usually undergo degradation in vitro in the presence of oxidizing agents such as H2O2. In vivo studies performed by Langer’s group have also demonstrated that It took 8 weeks for polydopamine implants to completely degrade.[26] It is quite a long time to wound healing. Therefore, the absorbed PDGF-BB most likely released from fibers due to rupture of hydrogen bond between PDGF-BB and polydopamine.……

**2.** **In the introduction the authors should discuss further why this specific PLGA was selected and discuss electrospinning of aliphatic polyesters, e.g.** **with regards to J. Mech. Behav. Biomed. Mater. 2017 (65) 724-733 and ACS Appl. Bio Mater. 2019 (2) 4258–4270.**

**Response: Thank you for your kind comments. The recent reports mentioned in the comments about the PLGA and electrospinning was quite interesting. And it has been cited in the revised manuscript to discuss why this specific PLGA was selected and discuss electrospinning of aliphatic polyesters.**

**Page 3:……**Poly[(rac-lactide)-co-glycolide] (PLGA), a FDA-approved polyester, has been widely used in the field of regenerative medicine due to its hydrolytic degradation and good controllability of macro properties. And electrospinning is a simple technique for the efficient production of micro to nano fibers from polymer solutions with high productivity and low cost. It was believed that electrospun fibers had the potential to achieve high drug loading and the ability to sustain drug release. Shih-Feng Chou studied the loading and release of tenofovir (TFV), a hydrophilic small molecule drug, into fibers of polycaprolactone (PCL) and poly (D,L-lactic-co-glycolic) acid (PLGA). The result showed that Sustained release of TFV depended on PLGA content. The PLGA fibers has outstanding sustained-release ability. Amy Contreras’ group encapsulated a photosensitizer (PS) in PCL/PLGA fibers in the PS inert state, so that the antibacterial function would be activated on-demand via a visible light source. This study successfully demonstrates the significant potential of PS-encapsulated electrospun fibers as photodynamically active biomaterial for antibiotic-free infection control. These studies fully demonstrated the advantages of PLGA electrospinning in drug sustained release.……
