# Supplementary material for: Polydopamine-assisted PDGF-BB immobilization on PLGA fibrous substrate enhances wound healing via regulating anti-inflammatory and cytokine secretion
Source: PLoS One. 2020 Sep 29;15(9):e0239366. doi: 10.1371/journal.pone.0239366 (PMC7523965; doi:10.1371/journal.pone.0239366)
Supplement: Attachment — Submitted filename: Responses to Reviewers.doc [file pone.0239366.s008.doc]

**NOTE:** The revised contents in the manuscript were labeled in RED. Our responses were written in BLUE just after each comment (BLACK) of the reviewers. The revised content of manuscript was briefly shown following our response.

First of all, we would like to express our great appreciation to the reviewers for their helpful comments and suggestions.

**The reviewer 1 and I agreed that the CD31 staining in Figure 7 doesn't mark the endothelium; however the green fluorescent staining is due to the auto-fluorescence of erytrocytes in the vessels. We are asking to provide a better staining of the vessels. CD31 antibody can be used together with alpha-SMA to define the vessels. Alternatively, vWF or Isolecti-B4 can be used to better identify the endothelial cells.**

**Response:** Thank you for your kind comments. The immunofluorescence images were replaced with immunohistochemical staining of CD31 with high magification. And the previous immunofluorescence images were settled in supporting information. positions of vascularization were point out with red arrows in merged images.

**Page 15-16:** Particularly, according to the result of CD31 staining (Fig 8), a lot of brown positive staining was observed in group of pDA/PLGA/PDGF-BB, which was believed to be neovascularization. PDA/PLGA/PDGF-BB substrate treated group showed a highly angiogenesis structure, which might be attributed to the facilitated penetration of PDGF-BB into deep dermal tissues and the prolonged residence of PDGF-BB conjugate. Most of the self-regeneration ability of skin was from basal layer , hair follicle and dermis . Therefore, the main task of repairing the full skin injury is to reconstruct the skin structure. It is recognized that extracellular matrix (ECM) deposition and vascularization are the most important in tissue regeneration including skin. PDGF has been accepted to stimulate the proliferation of various cells and the production of ECM . In addition, the PDGF stimulates human ﬁbroblasts to contract ﬂoating collagen matrices . In the process of tissue remodeling, PDGF helps to adjust the mitotic activity or the productivity of collagen in dermal ﬁbroblasts . To vascularization, it is worth noting that the effect of PDGF is weaker than that of FGF and VEGF and is not essential for the initial formation of blood vessels . But PDGF is particularly important in blood vessel maturation. The presence of the neovascularization indicated that the vessels were much more mature to take on more blood supply in group of pDA/PLGA/PDGF-BB. In *vivo* experiments have shown that PDGF is critical for the recruitment of pericytes, thereby increasing the structural integrity of blood vessels . In this study, it was found that the immobilized PDGF-BB greatly enhanced ECM maturation and vascularization, and provide the necessary ECM and vascular structure for skin regeneration.

**Fig 8. immunohistochemical staining of CD31.** Effects of the substitutes on neovascularization of wound area at day 7 after transplantation by examining the expression of CD31. (The bars=50 *μ*m)
